# Repeat hepatectomy is independently associated with favorable long‐term outcome in patients with colorectal liver metastases

**DOI:** 10.1002/cam4.872

**Published:** 2017-01-19

**Authors:** Christopher P. Neal, Gael R. Nana, Michael Jones, Vaux Cairns, Wee Ngu, John Isherwood, Ashley R. Dennison, Giuseppe Garcea

**Affiliations:** ^1^ Department of Hepatobiliary and Pancreatic SurgeryLeicester General HospitalGwendolen RoadLeicesterLE5 4PWUnited Kingdom

**Keywords:** Colorectal cancer, metastasectomy, prognosis

## Abstract

Up to three‐quarters of patients undergoing liver resection for colorectal liver metastases (CRLM) develop intrahepatic recurrence. Repeat hepatic resection appears to provide the optimal chance of cure for these patients. The aim of this study was to analyze short‐ and long‐term outcomes following index and repeat hepatectomy for CRLM. Clinicopathological data were obtained from a prospectively maintained database. Perioperative variables and outcomes were compared using the Chi‐squared test. Variables associated with long‐term survival following index and second hepatectomy were identified by Cox regression analyses. Over the study period, 488 patients underwent hepatic resection for CRLM, with 71 patients undergoing repeat hepatectomy. There was no significant difference in rates of morbidity (*P* = 0.135), major morbidity (*P* = 0.638), or mortality (*P* = 0.623) when index and second hepatectomy were compared. Performance of repeat hepatectomy was independently associated with increased overall and cancer‐specific survival following index hepatectomy. Short disease‐free interval between index and second hepatectomy, number of liver metastases >1, and resection of extrahepatic disease were independently associated with shortened survival following repeat resection. Repeat hepatectomy for recurrent CRLM offers short‐term outcomes equivalent to those of patients undergoing index hepatectomy, while being independently associated with improved long‐term patient survival.

## Introduction

Colorectal cancer (CRC) remains the second commonest cause of cancer death in the Western world 1. The liver is the organ to which CRC most frequently metastasizes, with 15–25% of patients having synchronous colorectal liver metastases (CRLM) at presentation and a further 25–50% ultimately developing CRLM following resection of the primary tumor 2. Hepatic resection offers the only hope of cure to patients with CRLM, providing 5‐year survival rates of 30–50% depending upon selection criteria 3, 4, 5. Unfortunately, cancer recurs in up to 75% of patients following hepatectomy, in the remnant liver and/or other sites 5. Over the past three decades, however, repeat hepatectomy has emerged as a viable therapy for recurrent CRLM 6, 7, 8, 9, 10. The rationale of repeat hepatectomy is supported by the observation that the liver is often the sole site of disease recurrence.

Although studies agree that repeat hepatectomy can be conducted with acceptable short‐term morbidity and mortality, they have produced conflicting results regarding the impact of repeat hepatectomy upon long‐term survival. While several large cohort studies have demonstrated repeat hepatectomy for recurrent CRLM to be associated with a survival benefit compared to patients undergoing a single hepatectomy 11, 12, other studies have yielded comparable long‐term results between the two groups 13, 14, 15, 16, 17. In addition, although improved survival after repeat hepatectomy has been shown in some studies, varying patient outcomes have been reported within individual studies. While attempts have been made to derive selection criteria for repeat hepatectomy, variables that predict recurrence in the repeat hepatectomy group remain to be clearly identified, with a plethora of significant prognostic variables identified in different studies 6, 7, 10, 15, 16, 18, 19, 20, 21, 22.

The aim of this study was to evaluate the short‐term and long‐term outcomes of patients undergoing repeat resection of CRLM, including the identification of factors associated with adverse long‐term survival following both index resection and repeat resection of CRLM. To the best of our knowledge, this is the first study to assess the impact of repeat hepatectomy on cancer‐specific survival following hepatectomy.

## Materials and Methods

### Study population and data collection

All patients undergoing hepatectomy for CRLM between January 2001 and December 2010 at a single tertiary referral hepatobiliary center were identified from a prospectively maintained database. These data were analyzed retrospectively, with any additional information gathered from medical records. The study was approved by our Institutional Review Board. Staging protocol prior to hepatectomy included contrast‐enhanced spiral computerized tomography of the chest, abdomen, and pelvis using an iodinated contrast agent and magnetic resonance imaging of the liver.

The criteria for acceptance for both index and repeat hepatectomy were: anatomically resectable hepatic disease on cross‐sectional imaging, the absence of distant, extra‐abdominal metastases (barring resectable lung metastases), and fitness for major surgery. Resectable hepatic disease for both index and repeat hepatectomy was defined by the ability to (1) spare at least two adjacent liver segments, (2) preserve vascular inflow and outflow, and (3) retain an adequate future liver remnant. All resections were carried out under low central venous pressure conditions. Liver parenchymal dissection was performed using a Sonoca ultrasonic dissector (Söring Medical, Quickborn, Germany), with hemostasis achieved using a combination of diathermy, argon beam coagulation, and suturing. If necessary, intermittent clamping of the portal triad (Pringle manoeuvre) was performed during parenchymal dissection, with periods of occlusion of up to 10 min alternating with 5 min release intervals. Ablative therapies (e.g., radiofrequency ablation) were not employed in any case. Following hepatic resection, patients were transferred to the intensive care unit (ICU) or high dependency unit (HDU) where an established clinical care pathway was followed.

Main short‐term postoperative outcome measures were morbidity, major morbidity (Clavien grade III‐V complications, i.e., requiring surgical, endoscopic, or radiological intervention, high dependency unit/intensive care unit management, or resulting in death) 23, and mortality. Morbidity and mortality were defined as occurring within 90 days of surgery. Resected specimens were assessed by a dedicated hepatobiliary pathologist. A positive (R1) margin was defined as <1 mm.

The follow‐up protocol consisted of a clinical review at 6 weeks post surgery, followed by clinical assessment, routine serum investigations (including liver function tests and serum carcinoembryonic antigen [CEA] level), and CT scan of the chest, abdomen, and pelvis—all performed once in 6 months for 2 years, then annually to at least 5 years post hepatectomy. Abnormal results during the surveillance period triggered further investigation. Development of symptoms prompted a review earlier than scheduled.

Long‐term outcome measures assessed were overall survival and cancer‐specific survival (as of August 2014). Causes of death were determined from case notes, computerized records, and death certificates. Patients who died within 90 days of hepatectomy (Clavien grade V complications) were excluded from long‐term survival analysis.

### Statistical analysis

Significant associations and differences between subgroups within the cohort were assessed using the Chi‐squared and Fisher's exact tests. Univariate prognostic significance of variables was determined by univariate Cox regression analysis, Kaplan–Meier analysis, and application of the log‐rank test. Multivariate analysis was performed through the entry of all variables with *P *<* *0.10 on univariate analysis into Cox proportional hazard regression analysis using a stepwise backward procedure. Statistical significance was defined as *P *<* *0.05. All statistical analyses were performed using Statistical Package for the Social Sciences 20.0^®^ (SPSS, Chicago, IL).

## Results

### Patient characteristics

During the study period, a total of 488 patients underwent hepatectomy for resection of CRLM, with all resections performed with curative intent. Recurrent disease developed in 338 patients following index hepatectomy (69.3%). Recurrence involved the liver in 231 patients (47.3%) and was liver‐only in 152 patients (31.1%). Seventy‐one patients (14.5%) developed operable recurrence and underwent a second hepatectomy. Twelve patients (2.5%) underwent a third hepatectomy and three patients (0.6%) underwent a fourth hepatectomy. Patient, tumor, and perioperative factors relating to index hepatectomy and first repeat hepatectomy are shown in Table 1 and Table 2. The median age at time of index hepatectomy was 64 years (mean age 63.4 years, range 26–85 years). There were 308 (63.1%) men and 180 (36.9%) women. At index hepatectomy, the median number of tumors on preoperative imaging was two (range 1–15). Tumor size ranged from 5 to 150 mm, with a median size of 35 mm. In total, 195 patients (40.0%) had a “major” resection (resection of three or more Couinaud segments). Two hundred and fourteen patients (43.9%) had systemic chemotherapy in the 6 months prior to their index liver resection and 195 patients (40.0%) received systemic chemotherapy following index metastasectomy.

**Table 1:** Comparison of disease and operative factors at index and repeat hepatectomy

Variable	Index resection (%) [*n* = 488]	Second resection(%) [*n* = 71]	*P*
Age at resection			
<65	252 (51.6%)	40 (56.3%)	0.459
≥65	236 (48.4%)	31 (43.7%)	
Gender			
Male	308 (63.1%)	49 (69.0%)	0.334
Female	180 (36.9%)	22 (31.0%)	
ASA grade			
1–2	411 (84.2%)	64 (90.1%)	0.192
3	77 (15.8%)	7 (9.9%)	
Primary tumor stage			
Node negative	177 (36.3%)	37 (52.1%)	**0.010**
Node positive	311 (63.7%)	34 (47.9%)	** **
Metastasis size			
≤50 mm	366 (75.0%)	59 (83.1%)	0.135
>50 mm	122 (25.0%)	12 (16.9%)	
Metastasis number			
1	210 (43.0%)	46 (64.8%)	**0.001**
>1	278 (57.0%)	25 (35.2%)	** **
CEA level			
≤200 ng/ml	458 (93.9%)	67 (94.4%)	1.00
>200 ng/ml	30 (6.1%)	4 (5.6%)	
Metastases distribution			
Unilobar	336 (68.9%)	57 (80.3%)	**0.049**
Bilobar	152 (31.1%)	14 (19.7%)	** **
Neoadjuvant chemotherapy			
Yes	214 (43.9%)	8 (11.3%)	**<0.001**
No	274 (56.1%)	63 (88.7%)	** **
Anatomical resection			
Yes	198 (40.6%)	14 (19.7%)	**0.001**
No	290 (59.4%)	57 (80.3%)	** **
Major hepatectomy			
Yes	195 (40.0%)	12 (16.9%)	**<0.001**
No	293 (60.0%)	59 (83.1%)	** **
Extrahepatic cancer resection			
Yes	14 (2.9%)	9 (12.7%)	**0.001**
No	474 (97.1%)	62 (87.3%)	

ASA, American Society of Anaesthesiologists; CEA, carcinoembryonic antigen. Bold value denotes *P *< 0.05.

**Table 2:** Outcomes following index and repeat hepatectomy

Variable	Index resection (%) [*n* = 488]	Second resection (%) [*n* = 71]	*P*
Resection margin			
RO	319 (65.4%)	44 (62.0%)	0.575
R1	169 (34.6%)	27 (38.0%)	
Operative duration			
<300 min	437 (89.5%)	68 (95.8%)	0.097
≥300 min	51 (10.5%)	3 (4.2%)	
Perioperative blood transfusion			
Yes	133 (27.3%)	22 (31.0%)	0.512
No	355 (72.7%)	49 (69.0%)	
Postoperative stay			
≤14 days	406 (83.2%)	59 (83.1%)	0.984
>14 days	82 (16.8%)	12 (16.9%)	
Any morbidity			
Yes	145 (29.7%)	15 (21.1%)	0.135
No	343 (70.3%)	56 (78.9%)	
Major morbidity			
Yes	50 (10.2%)	6 (8.5%)	0.638
No	438 (89.8%)	65 (91.5%)	

### Comparison of factors between index hepatectomy and first repeat hepatectomy groups

The number of metastases was significantly lower (*P *=* *0.001) and the distribution was more commonly unilobar in the repeat hepatectomy group (*P *=* *0.049) (Tables 1 and 2). In addition, both anatomical resections and major hepatectomies were significantly less frequent in the repeat resection group compared to the index hepatectomy population (*P *=* *0.001 and *P *<* *0.001, respectively). Concomitant resection of intra‐abdominal, extrahepatic disease was, however, significantly more common in the repeat resection group than the index resection group (12.7% vs. 2.9%, respectively, *P *=* *0.001).

There was no significant difference in resection margin status, operative duration, or perioperative blood transfusion requirement between the index resection and repeat hepatectomy groups. Morbidity was 29.7% in the index resection group and 21.1% in the repeat hepatectomy group (*P *=* *0.135). Major complications were encountered in 10.2% of the index hepatectomy group and 8.5% of the repeat resection group (*P *=* *0.638). Ninety‐day mortality was 2.0% (*n* = 10) in the index hepatectomy group and 0% in patients undergoing first repeat hepatectomy (*P *=* *0.623). For subsequent resections, 90‐day mortality was 8.3% (*n* = 1) following third hepatectomy and 0% following fourth hepatectomy.

### Identification of factors associated with long‐term survival following index hepatectomy

The median follow‐up period following index metastasectomy was 29.9 months (range: 4–189 months). The 3‐ and 5‐year overall survival rates following index metastasectomy were 48.0% and 30.1%, respectively (median 34.5 months, 95% confidence interval [CI]: 30.6–38.4 months). The 3‐ and 5‐year cancer‐specific survival rates following index metastasectomy were 51.5% and 35.1%, respectively (median 37.6 months, 95% CI: 32.3–42.9 months).

Patient and operative factors were assessed for their influence on long‐term survival following index hepatic resection. The following variables were significantly associated with poorer overall survival on univariate analysis (Table 3): age >65 years, largest metastasis size >50 mm, number of liver metastases >1, preoperative CEA >200 ng/mL, presence of bilobar disease, performance of major hepatectomy, positive (R1) microscopic margin, concomitant resection of extrahepatic, intra‐abdominal disease, perioperative blood transfusion, postoperative morbidity, and absence of repeat hepatectomy.

**Table 3:** Univariate Cox regression survival analyses for patients undergoing index metastasectomy (*n* = 478)

Variable	Patients(*n* = 478)	Overall survival		Cancer‐specific survival	
		Hazard ratio (95% CI)	*P*	Hazard ratio (95% CI)	*P*
Age: <65/≥65 years	248/230	1.287 (1.037–1.599)	**0.022**	1.260 (1.000–1.588)	0.050
Gender: male/female	299/179	0.876 (0.699–1.097)	0.249	0.783 (0.613–1.001)	0.051
Site of primary tumor: colon/rectum	235/243	1.002 (0.808–1.243)	0.986	0.953 (0.757–1.200)	0.682
Stage of primary tumor: node negative/node positive	174/304	1.178 (0.942–1.473)	0.152	1.167 (0.919–1.482)	0.206
Temporal presentation of metastases: DFI ≤12/>12 months	330/148	1.241 (0.977–1.576)	0.077	1.277 (0.987–1.653)	0.063
Diameter of liver metastases: ≤50/>50 mm	358/120	1.295 (1.017–1.648)	**0.036**	1.310 (1.012–1.695)	**0.040**
Number of liver metastases: 1/>1	209/269	1.542 (1.236–1.923)	**<0.001**	1.544 (1.218–1.957)	**<0.001**
Preoperative CEA: ≤200/>200 ng/mL	451/27	1.683 (1.119–2.532)	**0.012**	1.760 (1.149–2.697)	**0.009**
Bilobar disease: yes/no	144/334	1.476 (1.176–1.852)	**0.001**	1.466 (1.150–1.869)	**0.002**
Anatomical resection: yes/no	193/285	1.066 (0.856–1.327)	0.570	1.020 (0.806–1.291)	0.867
Major hepatectomy: yes/no	207/271	1.492 (1.203–1.852)	**<0.001**	1.455 (1.155–1.833)	**0.001**
Margin status: R0/R1	312/166	1.383 (1.107–1.727)	**0.004**	1.410 (1.112–1.786)	**0.004**
Extrahepatic metastasis resection: yes/no	14/464	2.858 (1.594–5.123)	**<0.001**	2.575 (1.361–4.872)	**0.004**
Perioperative blood transfusion: yes/no	126/352	1.431 (1.124–1.821)	**0.004**	1.459 (1.129–1.885)	**0.004**
Postoperative morbidity: yes/no	135/343	1.322 (1.038–1.684)	**0.024**	1.268 (0.977–1.644)	0.074
Chemotherapy within 6 months prior to liver resection: yes/no	206/272	1.120 (0.903–1.390)	0.303	1.111 (0.882–1.400)	0.372
Adjuvant chemotherapy: yes/no	195/283	0.878 (0.705–1.093)	0.245	0.956 (0.757–1.207)	0.706
Repeat hepatectomy: yes/no	71/407	0.586 (0.428–0.804)	**0.001**	0.656 (0.474–0.908)	**0.011**

Results for the following non‐significant variables (all *P *> 0.20) are not shown: American Society of Anaesthesiologists grade, Body Mass Index, Pringle manoeuvre duration, operative duration, perioperative blood transfusion, postoperative ITU stay, total hospital stay.

CI, confidence interval; DFI, disease‐free interval; CEA, carcinoembryonic antigen. Bold value denotes *P *< 0.05.

The following variables were associated with poorer cancer‐specific survival on univariate analysis (Table 3): largest metastasis size >50 mm, number of liver metastases >1, preoperative CEA >200 ng/mL, presence of bilobar disease, performance of major hepatectomy, positive (R1) microscopic margin, concomitant resection of extrahepatic disease, perioperative blood transfusion, and absence of repeat hepatectomy.

**Figure 1:**
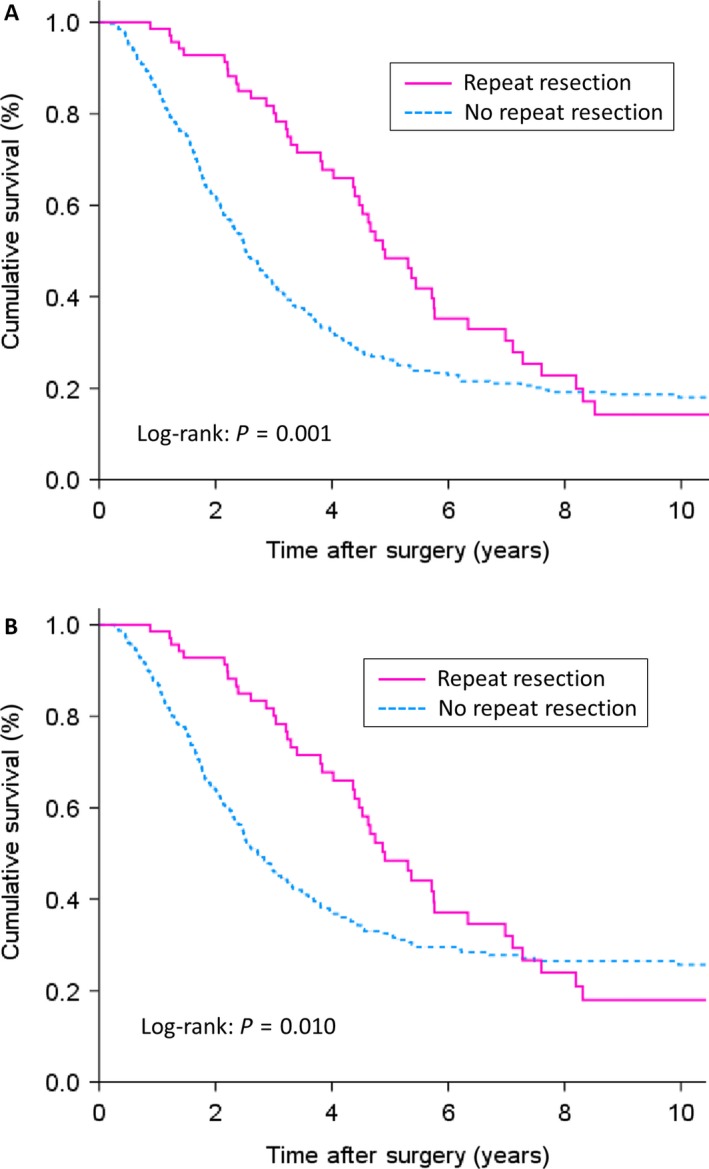
Kaplan–Meier survival curves for (**A)** overall survival and **(B)** cancer‐specific survival in 478 patients undergoing index hepatectomy according to the subsequent performance of repeat hepatectomy.

On multivariate analysis (Table 4), the following variables were independently associated with decreased overall survival following index metastasectomy: number of liver metastases >1 (HR 1.353, 95% CI: 1.033–1.774, *P *=* *0.028), preoperative CEA >200 ng/mL (HR 1.717, 95% CI: 1.134–2.601, *P *=* *0.011), positive (R1) microscopic margin (HR 1.330, 95% CI: 1.058–1.669, *P *=* *0.014), concomitant resection of extrahepatic disease (HR 2.736, 95% CI: 1.521–4.920, *P *=* *0.001), and absence of repeat hepatectomy (HR 0.636, 95% CI: 0.460–0.878, *P *=* *0.006). Three‐year and five‐year overall survival rates were 81.7% and 48.4% in patient undergoing repeat resection compared to 42.1% and 26.2% in patients undergoing index resection only (Fig. 1A). Patients in the repeat resection group had a median overall survival of 58.9 months; whereas, median survival in the index resection only group was 30.3 months.

**Table 4:** Multivariate Cox regression survival analyses for patients undergoing index metastasectomy (*n* = 478)

Variable	Patients(*n* = 478)	Overall survival		Cancer‐specific survival	
		Hazard ratio (95% CI)	*P*	Hazard ratio (95% CI)	*P*
Number of liver metastases: 1/>1	209/269	1.353 (1.033–1.774)	**0.028**	1.420 (1.105–1.824)	**0.006**
Preoperative CEA: ≤200/>200 ng/mL	451/27	1.717 (1.134–2.601)	**0.011**	1.723 (1.121–2.646)	**0.013**
Margin status: R0/R1	312/166	1.330 (1.058–1.669)	**0.014**	1.309 (1.027–1.669)	**0.030**
Extrahepatic metastasis resection: yes/no	14/464	2.736 (1.521–4.920)	**0.001**	2.489 (1.309–4.731)	**0.005**
Repeat hepatectomy: yes/no	71/407	0.636 (0.460–0.878)	**0.006**	0.672 (0.483–0.935)	**0.018**

CI, confidence interval; CEA, carcinoembryonic antigen. Bold value denotes *P *< 0.05.

On multivariate analysis, the same five variables were also found to exert independent prognostic significance in relation to cancer‐specific survival following index metastasectomy (Table 4). Specifically, number of liver metastases >1 (HR 1.420, 95% CI: 1.105–1.824, *P *=* *0.006), preoperative CEA >200 ng/mL (HR 1.723, 95% CI: 1.121–2.646, *P *=* *0.013), positive (R1) microscopic margin (HR 1.309, 95% CI: 1.027–1.669, *P *=* *0.030), concomitant resection of extrahepatic disease (HR 2.489, 95% CI: 1.309–4.731, *P *=* *0.005), and absence of repeat hepatectomy (HR 0.672, 95% CI: 0.483–0.935, *P *=* *0.018) were independently associated with shortened cancer‐specific survival. Three‐year and five‐year cancer‐specific survival rates were 81.7% and 48.4% in patients undergoing repeat resection, compared to 46% and 32.1% in patients undergoing index resection only (Fig. 1B). Patients in the repeat resection group had a median overall survival of 58.9 months, whereas, median survival in the index resection only group was 32.8 months.

Survival analyses confirmed that patients undergoing repeat hepatic resection had significantly greater overall and cancer‐specific survival than patients with inoperable recurrence but significantly shorter overall and cancer‐specific survival than patients who remained disease‐free following index resection (all *P* < 0.01, log‐rank test).

Survival analyses were then repeated to compare survival of the single resection group with survival of the repeat hepatectomy group from time of second hepatic resection. Regarding overall survival, 3‐year and 5‐year survival rates were 42.1% and 26.2% in patients undergoing index resection only and 52.6% and 21.8% in patients undergoing repeat resection. Median survival was 30.3 months in the single resection group and 38.2 months in the repeat resection group (*P *=* *0.811, log‐rank test). Regarding cancer‐specific survival, 3‐year and 5‐year survival rates were 46.0% and 32.1% in patients undergoing single resection only and 70.7% and 23.9% in patients undergoing repeat resection. Median survival was 32.8 months in the single resection group and 38.2 months in the repeat resection group (*P *=* *0.782, log‐rank test). These data confirmed that survival of the repeat resection group from time of second resection was equivalent to survival of the patient group undergoing a single resection. Following third hepatectomy, median overall survival and median cancer‐specific survival were 36.1 months.

### Identification of factors associated with survival following second hepatectomy

Survival analyses were then repeated to analyze the influence of variables upon long‐term survival following second hepatectomy, assessing patient and perioperative factors relating to both index and repeat hepatectomy. On univariate analysis (Table 5), patients age >65, disease‐free interval ≤12 months between index and repeat hepatectomy, and number of liver metastases at repeat resection >1 were significantly associated with shorter overall survival following repeat metastasectomy. These same variables, in addition to concomitant resection of extrahepatic disease at repeat hepatectomy, were also significantly associated with decreased cancer‐specific survival following hepatectomy on univariate analysis.

**Table 5:** Univariate Cox regression survival analyses for all patients undergoing second hepatectomy (*n* = 71)

Variable	Patients(*n* = 71)	Overall survival		Cancer‐specific survival	
		Hazard ratio (95% CI)	*P*	Hazard ratio (95% CI)	*P*
(a) Factors relating to index resection					
Age: <65/≥65 years	48/23	1.915 (1.031–3.557)	**0.040**	1.974 (1/057–3.687)	**0.033**
Gender: male/female	48/23	1.241 (0.657–2.346)	0.506	1.325 (0.696–2.521)	0.392
Site of primary tumor: colon/rectum	37/34	0.778 (0.432–1.402)	0.404	0.721 (0.395–1.316)	0.286
Stage of primary tumor: node positive/negative	34/37	1.465 (0.809–2.651)	0.207	1.357 (0.739–2.491)	0.325
Disease‐free interval: ≤12/>12 months	58/13	1.753 (0.740–4.153)	0.202	1.640 (0.691–3.896)	0.262
Diameter of liver metastases: ≤50/>50 mm	58/13	1.291 (0.594–2.807)	0.518	1.315 (0.604–2.864)	0.490
Number of liver metastases: 1/>1	29/42	1.234 (0.657–2.315)	0.513	1.231 (0.646–2.346)	0.528
Preoperative CEA: ≤200/>200 ng/mL	67/4	1.480 (0.523–4.189)	0.460	1.480 (0.523–4.189)	0.460
Bilobar disease: yes/no	22/49	1.542 (0.825–2.883)	0.175	1.563 (0.834–2.930)	0.164
Anatomical resection: yes/no	17/54	1.159 (0.595–2.259)	0.664	1.215 (0.622–2.373)	0.569
Major hepatectomy: yes/no	18/53	1.462 (0.737–2.899)	0.277	1.353 (0.678–2.695)	0.391
Margin status: R0/R1	50/21	1.460 (0.736–2.896)	0.278	1.424 (0.716–2.832)	0.313
Postoperative morbidity: yes/no	10/61	1.391 (0.583–3.318)	0.457	1.425 (0.596–3.406)	0.425
Neoadjuvant chemotherapy: yes/no	36/35	1.202 (0.662–2.181)	0.545	1.166 (0.633–2.146)	0.622
Adjuvant chemotherapy: yes/no	34/37	0.782 (0.434–1.408)	0.412	0.717 (0.392–1.312)	0.281
(b) Factors relating to second resection					
Disease‐free interval: ≤12/>12 months	46/25	2.004 (1.088–3.690)	**0.026**	2.237 (1.209–4.149)	**0.010**
Diameter of liver metastases: ≤50/>50 mm	59/12	0.935 (0.367–2.378)	0.887	0.973 (0.382–2.477)	0.954
Number of liver metastases: 1/>1	46/25	2.068 (1.125–3.800)	**0.019**	1.881 (1.015–3.485)	**0.045**
Preoperative CEA: ≤200/>200 ng/mL	67/4	1.129 (0.272–4.694)	0.867	1.129 (0.272–4.694)	0.867
Bilobar disease: yes/no	14/57	0.844 (0.404–1.761)	0.651	0.891 (0.425–1.865)	0.759
Anatomical resection: yes/no	15/56	1.014 (0.471–2.184)	0.972	1.101 (0.510–2.377)	0.807
Major hepatectomy: yes/no	15/56	0.978 (0.454–2.107)	0.955	1.060 (0.491–2.289)	0.882
Margin status: R0/R1	44/27	1.477 (0.795–2.745)	0.217	1.640 (0.860–3.127)	0.133
Extrahepatic metastasis resection: yes/no	9/62	1.845 (0.764–4.454)	0.173	2.218 (1.021–4.435)	**0.044**
Postoperative morbidity: yes/no	15/56	0.951 (0.454–1.992)	0.894	0.851 (0.392–1.846)	0.683
Neoadjuvant chemotherapy: yes/no	8/63	0.951 (0.400–2.261)	0.910	1.033 (0.434–2.455)	0.942
Adjuvant chemotherapy: yes/no	20/51	1.077 (0.574–2.021)	0.818	1.098 (0.583–2.069)	0.771

Results for the following nonsignificant variables (all *P *> 0.20) are not shown: American Society of Anaesthesiologists grade, Body Mass Index, Pringle manoeuvre duration, operative duration, perioperative blood transfusion, postoperative ITU stay, total hospital stay.

CI, confidence interval; CEA, carcinoembryonic antigen. Bold value denotes *P *< 0.05.

On multivariate analysis (Table 6), disease‐free interval ≤12 months between index and repeat hepatectomy (HR 2.632, 95% CI: 1.399–4.950, *P *=* *0.003) and number of liver metastases at repeat resection >1 (HR 2.903, 95% CI: 1.523–5.533, *P *=* *0.001) were independently associated with shortened overall survival following repeat resection. Regarding cancer‐specific survival, disease‐free interval ≤12 months between index and repeat hepatectomy (HR 3.279, 95% CI: 1.684–6.410, *P *<* *0.001), number of liver metastases at repeat resection >1 (HR 2.359, 95% CI: 1.213–4.587, *P *=* *0.011), and concomitant resection of extrahepatic metastatic disease at repeat hepatectomy (HR 2.566, 95% CI: 1.327–4.962, *P *=* *0.005) were independently associated with shortened survival following second hepatectomy.

**Table 6:** Multivariate Cox regression survival analyses for all patients undergoing second hepatectomy (*n* = 71)

Variable	Patients(*n* = 71)	Overall survival		Cancer‐specific survival	
		Hazard ratio (95% CI)	*P*	Hazard ratio (95% CI)	*P*
Factors relating to second resection					
Disease‐free interval: ≤12/>12 months	46/25	2.632 (1.399–4.950)	**0.003**	3.279 (1.684–6.410)	**<0.001**
Number of liver metastases: 1/>1	46/25	2.903 (1.523–5.533)	**0.001**	2.359 (1.213–4.587)	**0.011**
Extrahepatic metastasis resection: yes/no	9/62		** **	2.566 (1.327–4.962)	**0.005**

CI, confidence interval; CEA, carcinoembryonic antigen. Bold value denotes *P *< 0.05.

## Discussion

This study confirms that repeat hepatic resection for recurrent CRLM is safe, with short‐term outcomes equivalent to those reported following index resection. Second hepatectomy was performed in 14.5% (71/488) of patients undergoing index CRLM resection in this series, which is in line with the median 13.5% repeat resection rate (range 7–32%) reported in a recent systematic review 24. Compared to initial liver resection, liver metastases at the time of second hepatectomy were significantly more likely to be solitary and unilobar in distribution and, in line with this, major hepatectomy was performed less frequently during second resections. These observations are in agreement with the results of a recent meta‐analysis of published studies 25.

Despite the increased surgical demands inherent in repeat hepatic resection, resulting from intra‐abdominal adhesions and altered anatomy in the hypertrophied liver remnant, we found no significant difference in operative duration, margin status, blood transfusion requirement, or postoperative stay between the index resection and second hepatectomy groups. Significantly, both morbidity and major morbidity rates after second hepatectomy (21.1% and 8.5%, respectively) were, in fact, marginally lower than those after index hepatectomy at our center. The morbidity we report (21.1%) is in line with the literature, with a median morbidity rate of 23% (range 12–57%) after second hepatectomy recently reported 24. Moreover, the mortality rate of 0% in the current series for first repeat hepatectomy agrees with available data and further supports the concept of repeat surgery for CRLM.

Due to the widely accepted survival advantage of index liver resection for potentially operable CRLM over nonoperative treatment, no controlled trial of repeat hepatectomy has ever been performed, with studies relying on the analysis of populations undergoing primary and index resection for CRLM in order to attempt to delineate its role. Studies to date, however, have yielded conflicting results regarding the influence of repeat hepatectomy for CRLM upon long‐term survival 11, 12, 13, 14, 15, 16, 17. A notable finding of this study was that repeat hepatectomy was significantly associated with improved overall and cancer‐specific survival following metastasectomy, along with established prognostic factors including tumor number and margin status. Furthermore, this significance was maintained on multivariate analyses that included other known clinicopathological risk factors for long‐term survival. These data agree with the conclusions of a recent meta‐analysis of studies assessing repeat hepatectomy for recurrent CRLM 25. While this earlier analysis found no significant difference in overall survival between the single resection and repeat resection groups when all studies were evaluated, repetition of analyses to include just high‐ quality studies 11, 12, 14, 15 or just studies with greater than 500 cases demonstrated that repeat resection was significantly associated with improved overall survival following metastasectomy. In agreement with this, Saiura et al. recently found the performance of repeat resection to be independently associated with prolonged overall survival following metastasectomy 26. The current data further support this independent association of repeat resection with improved outcome and demonstrate, for the first time, that the association also extends to cancer‐specific survival. Together, these data support the use of repeat resection for CRLM. The equivalent survival we report following index resection in patients undergoing one resection and following second hepatectomy in patients with operable recurrence supports the previously raised notion that repeat resection essentially “resets the oncological clock” in patients with resectable CRLM 24.

As for index hepatectomy, there remains significant variability in patient outcome following repeat hepatectomy and we therefore also sought to identify factors associated with outcome, specifically following repeat resection. This analysis demonstrated that only factors relating to the repeat resection exerted independent prognostic significance. Specifically, short interval between index and repeat resection, more than one metastasis at repeat resection, and the resection of extrahepatic disease at time of repeat resection were independently associated with shortened survival following repeat resection. Of note, a recent systematic review of repeat hepatectomy series reported that studies had identified 17 prognostic factors associated with adverse outcome following repeat resection, but with no one factor reported by all studies 24. Without doubt, this variation is due, in part, to the relatively small number of repeat resections in individual studies. They concluded that metastasis size, disease‐free interval >12 months, and margin status were the most reported prognostic factors; findings that are in line with current study. More recently, a meta‐analysis of published studies reported the following factors to be significantly associated with adverse outcome following repeat resection, all relating to the second rather than the index operation: disease‐free interval, metastasis number, metastasis size, unilobar disease, concomitant extrahepatic metastases, and margin status 25. Of note, the independent factors identified in this study are among this number.

## Conclusions

In summary, this study confirms the value of repeat hepatic resection in patients with CRLM, providing short‐term outcomes equivalent to index resection and being independently associated with improved long‐term survival, in relation to both overall and cancer‐specific survival. Further large studies are needed to confirm these results and to enable more accurate delineation of prognostic variables associated with poor survival following repeat metastasectomy, so that potential patient benefit may be more accurately assessed.

## Conflict of Interest

None declared.

## References

[1] Siegel, R. , D. Naishadham , and A. Jemal . 2013 Cancer statistics, 2013. CA Cancer 63:11–30. 10.3322/caac.21166 23335087

[2] McMillan, D. C. , and C. S. McArdle . 2007 Epidemiology of colorectal liver metastases. Surg. Oncol. 16:3–5. 17493802 10.1016/j.suronc.2007.04.008

[3] Fong, Y. , J. Fortner , R. L. Sun , M. F. Brennan , and L. H. Blumgart . 1999 Clinical score for predicting recurrence after hepatic resection for metastatic colorectal cancer: analysis of 1001 consecutive cases. Ann. Surg. 230:309–318. 10493478 10.1097/00000658-199909000-00004 PMC1420876

[4] Malik, H. Z. , K. R. Prasad , K. J. Halazun , A. Aldoori , A. Al‐Mukhtar , D. Domez , et al. 2007 Preoperative prognostic score for predicting survival after hepatic resection for colorectal liver metastases. Ann. Surg. 246:806–814. 17968173 10.1097/SLA.0b013e318142d964

[5] Simmonds, P. C. , J. N. Primrose , J. L. Colquitt , O. J. Garden , G. J. Poston , and M. Rees . 2006 Surgical resection of hepatic metastases from colorectal cancer: a systematic review of published studies. Brit. J. Cancer 94:982–999. 16538219 10.1038/sj.bjc.6603033 PMC2361241

[6] Adam, R. , H. Bismuth , D. Castaing , F. Waechter , F. Navarro , A. Abascal , et al. 1997 Repeat hepatectomy for colorectal liver metastases. Ann. Surg. 225:51–60. 8998120 10.1097/00000658-199701000-00006 PMC1190605

[7] de Jong, M. C. , S. C. Mayo , C. Pulitano , S. Lanella , D. Ribero , J. Strub , et al. 2009 Repeat curative intent liver surgery is safe and effective for recurrent colorectal liver metastasis: results from an international multi‐institutional analysis. J. Gastrointest. Surg. 13:2141–2151. 19795176 10.1007/s11605-009-1050-0

[8] Fong, Y. , L. H. Blumgart , A. Cohen , J. Fortner , and M. F. Brennan . 1994 Repeat hepatic resections for metastatic colorectal cancer. Ann. Surg. 220:657–662. 7979614 10.1097/00000658-199411000-00009 PMC1234454

[9] Fortner, J. G. 1988 Recurrence of colorectal cancer after hepatic resection. Am. J. Surg. 155:378–382. 3344897 10.1016/s0002-9610(88)80086-2

[10] Petrowsky, H. , M. Gonen , W. Jarnagin , M. Lorenz , R. DeMatteo , S. Heinrich , et al. 2002 Second liver resections are safe and effective treatment for recurrent hepatic metastases from colorectal cancer: a bi‐institutional analysis. Ann. Surg. 235:863–871. 12035044 10.1097/00000658-200206000-00015 PMC1422517

[11] Shaw, I. M. , M. Rees , F. K. Welsh , S. Bygrave , and T. G. John . 2006 Repeat hepatic resection for recurrent colorectal liver metastases is associated with favourable long‐term survival. Brit. J. Surg. 93:457–464. 16555242 10.1002/bjs.5323

[12] Wicherts, D. A. , R. J. de Haas , C. Salloum , P. Andreani , G. Pascal , D. Sotirov , et al. 2013 Repeat hepatectomy for recurrent colorectal metastases. Brit. J. Surg. 100:808–818. 23494765 10.1002/bjs.9088

[13] Jonsson, K. , G. Grondahl , M. Salo , B. Tingstedt , and R. Anderson . 2012 Repeated Liver Resection for Colorectal Liver Metastases: A Comparison with Primary Liver Resections concerning Perioperative and Long‐Term Outcome. Gastroenterol. Res. Pract. 2012:568214. 22973305 10.1155/2012/568214 PMC3437631

[14] Rolff, H. C. , D. Calatayud , P. N. Larsen , and A. Wettergren . 2012 Good results after repeated resection for colorectal liver metastases. Dan. Med. J. 59:A4373. 22293047

[15] Sa Cunha, A. , C. Laurent , A. Rault , P. Couderc , E. Rullier , and J. Saric . 2007 A second liver resection due to recurrent colorectal liver metastases. Arch. Surg. 142:1144–1149. 18086980 10.1001/archsurg.142.12.1144

[16] Takahashi, S. , K. Inoue , M. Konishi , T. Nakagouri , and T. Kinoshita . 2003 Prognostic factors for poor survival after repeat hepatectomy in patients with colorectal liver metastases. Surgery 133:627–634. 12796730 10.1067/msy.2003.151

[17] Treska, V. , T. Skalicky , V. Liska , and J. Ferda . 2007 Repeated procedures for colorectal liver metastases. Hepatogastroenterology 54:1775–1778. 18019716

[18] Andreou, A. , A. Brouquet , E. K. Abdalla , T. A. Abdalla , S. A. Curley , and J. N. Vauthey . 2011 Repeat hepatectomy for recurrent colorectal liver metastases is associated with a high survival rate. HPB (Oxford) 13:774–782. 21999590 10.1111/j.1477-2574.2011.00370.x PMC3238011

[19] Brachet, D. , E. Lermite , A. Rouquette , G. Lorimier , A. Hamy , and J. P. Arnaud . 2009 Prognostic factors of survival in repeat liver resection for recurrent colorectal metastases: review of sixty‐two cases treated at a single institution. Dis. Colon Rectum 52:475–483. 19333049 10.1007/DCR.0b013e31819d12bc

[20] Nishio, H. , Z. Z. Hamady , H. Z. Malik , S. Fenwick , R. Prasad , G. J. Toogood , et al. 2007 Outcome following repeat liver resection for colorectal liver metastases. Eur. J. Surg. Oncol. 33:729–734. 17258883 10.1016/j.ejso.2006.07.005

[21] Suzuki, S. , T. Sakaguchi , Y. Yokoi , K. Kurachi , K. Okamoto , T. Okumura , et al. 2001 Impact of repeat hepatectomy on recurrent colorectal liver metastases. Surgery 129:421–428. 11283532 10.1067/msy.2001.112486

[22] Thelen, A. , S. Jonas , C. Benckert , G. Schumacher , E. Lopez‐Hanninen , B. Rudolph , et al. 2007 Repeat liver resection for recurrent liver metastases from colorectal cancer. Eur. J. Surg. Oncol. 33:324–328. 17112697 10.1016/j.ejso.2006.10.016

[23] Dindo, D. , N. Demartines , and P. A. Clavien . 2004 Classification of surgical complications: a new proposal with evaluation in a cohort of 6336 patients and results of a survey. Ann. Surg. 240:205–213. 15273542 10.1097/01.sla.0000133083.54934.ae PMC1360123

[24] Lam, V. W. , T. Pang , J. M. Laurence , E. Johnston , M. J. Hollands , H. C. Pleass , et al. 2013 A systematic review of repeat hepatectomy for recurrent colorectal liver metastases. J. Gastrointest. Surg. 17:1312–1321. 23525970 10.1007/s11605-013-2186-5

[25] Luo, L. X. , Z. Y. Yu , J. W. Huang , and H. Wu . 2014 Selecting patients for a second hepatectomy for colorectal metastases: an systemic review and meta‐analysis. Eur. J. Surg. Oncol. 40:1036–1048. 24915859 10.1016/j.ejso.2014.03.012

[26] Saiura, A. , J. Yamamoto , R. Koga , Y. Takahashi , M. Takahashi , Y. Inoue , et al. 2014 Favorable outcome after repeat resection for colorectal liver metastases. Ann. Surg. Oncol. 21:4293–4299. 24962942 10.1245/s10434-014-3863-7

